# TINU: A Multisystemic Inflammatory Disorder—Case Report and Literature Review

**DOI:** 10.1155/2024/3909755

**Published:** 2024-04-10

**Authors:** Juan Montejo-Hernández, Jorge Rico-Fontalvo, Jose Cabrales, Shuchi Anand, María Cristina Martínez-Ávila, Claudia Duran-Merino, Luis Arias-Restrepo, Camilo Andrés Gómez Duran

**Affiliations:** ^1^Colombian Association of Nephrology and Hypertension, Bogotá, Colombia; ^2^Division of Nephrology, Stanford Healthcare, Stanford University School of Medicine, Palo Alto, Colombia; ^3^Epidemiology and Public Health, Polytechnic University of Nicaragua, Managua, Nicaragua; ^4^San Diego Ophthalmology Clinic, Medellín, Colombia; ^5^Department of Pathology, University of Antioquia, Medellín, Colombia; ^6^CES University, Medellín, Colombia

## Abstract

*Background*. The syndrome of tubulointerstitial nephritis and uveitis (TINU) is a rare oculorenal condition, mainly seen in children and women. The underlying cause of this disease is unknown. *Case Presentation*. We report a 24-year-old male without any past medical history, diagnosed with bilateral uveitis and azotemia. Biopsy revealed tubulointerstitial nephritis, consistent with TINU syndrome. Fluorescein angiogram revealed peripheral retinal vasculitis. *Discussion*. TINU is a rare disorder that needs to be distinguished from sarcoidosis, Sjogren's disease, and tuberculosis. Treatment is indicated in patients with progressive renal insufficiency, consisting of steroid therapy. Most patients recover kidney function. Its early recognition is important to offer the best chance of organ preservation.

## 1. Background

Tubulointerstitial nephritis and uveitis syndrome, TINU, or Dobrin syndrome, is a multisystemic inflammatory condition that is rare. In the year 1975, Dobrin et al. [1] first reported 2 cases of anterior uveitis and acute eosinophilic interstitial nephritis associated with myeloid sarcoma. Since then, at least 300 cases have been reported in the literature, mainly in pediatric nephrology and in ophthalmology journals. It is defined as the presence of tubulointerstitial nephritis in the absence of systemic disease [2].

We present a case of idiopathic biopsy-proven TINU syndrome in a 24-year-old male, with the unusual finding of peripheral retinal vasculitis.

## 2. Case Presentation

A 24-year-old male presented to the emergency department complaining of one month of bilateral blurry vision, conjunctival erythema, and ocular pain. He was initially thought to have viral conjunctivitis, for which he was prescribed lubricant drops and sent home. Given the lack of improvement, he was seen in the ophthalmology clinic, found to have non-granulomatous uveitis and vitritis, with inferior elevation of the retina. Hence, he was prescribed prednisolone drops every four hours plus tropicamide drops.

Laboratory exams done before the ophthalmology visit, with his primary care physician, revealed a negative IgM and IgG for toxoplasmosis and negative human immunodeficiency virus (HIV) and syphilis serologies. Testing for rheumatoid factor (RF) was also negative. Subsequent routine laboratory testing revealed a creatinine of 1.93 mg/dL, blood urea nitrogen (BUN) of 28 mg/dL, white blood cell count (WBC) count of 5110 per microliter, hemoglobin (Hb) of 13.7 g/dL, and 293,000 platelets per microliter. The urinalysis was bland, with no protein or blood. Erythrocyte sedimentation rate was 9 mm/hr. Given his persistent symptoms, he was treated with deflazacort taper, starting with the dose of 30 mg twice daily for a week, with 15 mg decreases weekly for four weeks.

**Figure 1:**
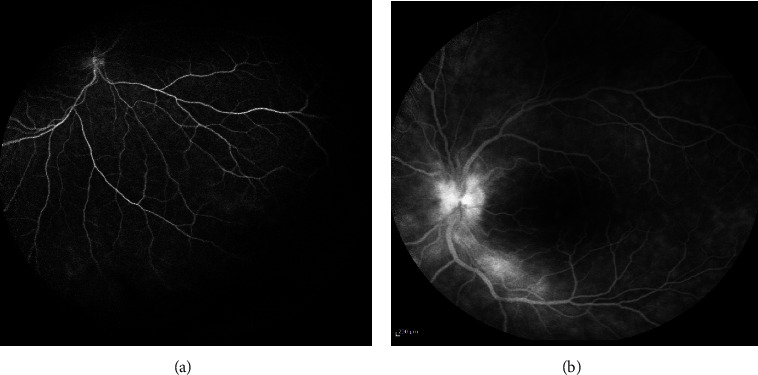
Fluorescein angiogram revealing the following: (a) peripheral retinal vasculitis in the right eye; (b) papillitis and macular edema of the left eye.

Given his partial resolution of symptoms, he was evaluated by neuro-ophthalmology and was diagnosed with bilateral non-granulomatous panuveitis. He underwent a fluorescein angiography which showed peripheral retinal vasculitis in the right eye and left-sided papillitis and macular edema, as shown in Figure 1. Due to his elevated creatinine found incidentally, he was referred to the nephrology clinic.

**Figure 2:**
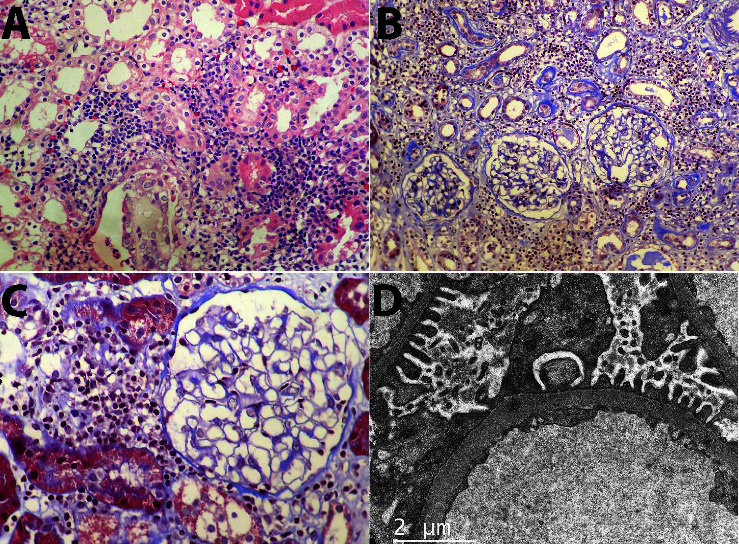
(A) Interstitial mononuclear inflammatory infiltrate, of lymphocytic predominance; also seen is tubular involvement with destruction (tubulitis). Hematoxylin-Eosin, 400x. (B) The inflammatory infiltrate expands the interstitium, separating the tubules, some of which show atrophic changes, evidenced by a thickened and irregular basement membrane, highlighted in blue in this trichrome stain, 200x. (C) Normal glomeruli, without increase in cellularity nor mesangial or capillary wall damage. To the left of the glomerulus, there is the inflammatory infiltrate, limited by Bowman's capsule. Trichrome stain, 400x. (D) Electron microscopy (EM) revealing normal glomeruli, with normal podocytes, basal membrane, and endothelium. EM, 2500x.

At his initial visit with nephrology, his vital signs were within normal limits, and additional studies were obtained, revealing proteins in urine 24-hour were 338 mg, creatinine clearance was 75 ml/min/1.73m2, antinuclear antibodies (ANA) test positive 1:640, c-ANCA and p-ANCA negative, extractable nuclear antigen (ENA) panel negative, and normal complements. Markers of tubular damage were present (urinary beta-2 microglobulin 32.33 mg/dL, α1 microglobulin 161.51 mg/L, N-acetylglucosaminidase (NAG) was not performed due to its lack of sufficient specificity). A kidney biopsy was ordered, which revealed interstitial edema and multiple foci of mononuclear inflammatory cells, with tubular epithelial exocytosis (tubulitis), several zones of fibrosis, and tubular atrophy in approximately 20% of the sample (Figure 2). Immunofluorescence was negative for IgA, IgG, IgM, C3, and C1q.

He was then treated with an oral prednisolone taper starting at 50 mg/day, with a plan for chronic therapy for 3 months, but the patient abruptly stopped the medication at 30 mg, as he was having mood swings. Afterward, immunosuppressive therapy was indicated until symptoms improvement.

Follow-up testing revealed his creatinine was improving to 1.24 mg/dL after steroid therapy, and proteinuria decreased to 190 mg of protein per day in a follow-up 24-hour urine collection. His uveitis symptoms improved significantly; he will be followed up conservatively in the nephrology and ophthalmology clinic (Table 1).

### 2.1. Epidemiology and Etiologic Factors

There are several proposed risk factors, including younger age, female gender, and genetic predisposition. Mandeville et al. suggested that HLA A-2 and HLA A-24 were antigens of importance in association with this disorder in Japanese subjects, as they were found in 75% of the patients diagnosed with TINU [2]. The estimated prevalence of this rare condition is usually less than 2% across the uveitis services, but the data are limited [3].

Several triggers for this disease have been found; TINU has been diagnosed in patients with evidence of recent Epstein–Barr virus infection (EBV) and patients with hepatic tuberculosis and even chlamydial infections [4]. It is thought to be an inflammatory response to an infectious trigger, although chemical and pharmacological triggers have also been reported, including non-steroidal anti-inflammatory drugs, acetaminophen, codeine, and the herbal formulation Goreisan in one case report [4]. Haplotypes that have been found to be associated with this condition are HLA-DQA1^*∗*^01, HLA-DQB1^*∗*^05, and HLA-DRB1^*∗*^01 [5]. In one case report, a mother and her son were both affected by the syndrome, reflecting on the genetic component of the disease [6].

### 2.2. Pathogenesis and Pathophysiology

Most of the cases of TINU are either autoimmune or infectious in etiology; although there has been an association with infectious triggers, there is no evidence of infection at the tissue level. Hence, the consensus is that the disease is autoimmune [4]. A study done by Tan et al. [7] demonstrated that there were antibodies directed against modified C-reactive protein (CRP) in 9 patients with TINU, in higher concentrations compared to control patients that had other forms of kidney disease [5, 7]. They later showed that these antibodies are in both the kidney and in ocular tissue with modified CRP, suggesting that the latter might be a target antigen for TINU [4].

Given the usual monocytic and lymphocytic interstitial infiltrate seen in the biopsy, it is thought that cellular immunity also plays a role in the pathophysiology of this disease. The association with HLA genotypes also suggests cellular immunity is of importance. The immunopathogenic model proposes that a microbial or chemical trigger interacts with Toll-like receptors in antigen-presenting cells (APC), which initiates the primary phase of the innate immune response. The antigen later associates with HLA peptides, which enhances transcription of immune response and proinflammatory genes. Eventually, antigen-presenting T cells proliferate and lead to cytokine production, inflammation, and B cell activation, enhancing a humoral response as well, that leads to autoimmune inflammation and tissue injury [4].

### 2.3. Clinical Manifestations and Diagnosis

Mandeville et al. [2] proposed a diagnostic strategy for these patients; the diagnosis usually requires the presence of both ocular and kidney involvement, in the absence of systemic disease [2, 3, 8]. Per these criteria, definite TINU syndrome is characterized by acute interstitial nephritis (AIN) in the setting of typical uveitis, probable TINU is defined as AIN diagnosed by biopsy and atypical uveitis or clinically diagnosed AIN with typical uveitis, and possible is defined as clinically diagnosed AIN with atypical uveitis [2, 4]. In addition to uveitis and interstitial nephritis, some patients might present systemic manifestations, such as fever, fatigue, and anorexia. When associated with drug exposure, clinical history is of great importance.

A case series of four patients published by Lopes et al. [8] revealed that the laboratory abnormalities found in their patients were like the ones described by Mandeville et al. including abnormal kidney function, evidence of tubular dysfunction with elevated urinary beta-2 microglobulin, proteinuria, hematuria, glycosuria (Fanconi's syndrome), urinary eosinophils, and/or WBC casts [2, 8]. Most of the patients present in one of two ways; like our patient, many present with uveitis symptoms to the ophthalmologist and will have laboratory testing revealing azotemia. The other subset of patients usually presents with azotemia of unknown cause, possibly following a flu-like illness [4]. Patients usually present with proteinuria of less than 1 gram per day, mainly representing tubular proteins. Increased urinary excretion of beta-2 microglobulin and urinary NAG is frequently seen in these patients [4]. Additional laboratory findings include elevated CRP and erythrocyte sedimentation rate (ESR), although non-specific, like the abovementioned. It has been proposed that the mucoprotein Krebs von den Lunge-6 (KL-6) may be useful as a serologic marker for the diagnosis of TINU, as it is elevated in TINU, but not in other forms of uveitis [4].

Differential diagnosis usually includes conditions that usually have the coexistence of uveitis and interstitial nephritis, which include Sjogren's syndrome, sarcoidosis, granulomatosis with polyangiitis, nephronophthisis, tuberculosis, lupus, and Behcet's disease. Differentiating from sarcoidosis can be challenging, as sarcoid disease presents with both uveitis and interstitial disease as well.

As in our patient, uveitis is typically non-granulomatous and bilateral, and vitreous or choroidal involvement is not uncommon [8]. Usually, kidney involvement precedes eye symptoms, although both occur simultaneously in around 15% of the cases, which makes the diagnosis of the disease challenging in many of the cases [8].

### 2.4. Renal Histopathology

The most typical finding in TINU regarding its histopathology is a lymphocytic interstitial infiltrate, composed mainly of CD3-positive lymphocytes, with fewer plasma cells and macrophages, associated with tubulitis [4, 9]. Initially, patients may have eosinophilic infiltration, which eventually disappears; subsequent fibrosis and less inflammation occur as the disease progresses. The IF, as seen in our patient, is usually without glomerular or tubular staining [8]. Figure 2 shows the histopathologic findings of our patient, in which there is evidence of monocytic infiltration with lymphocytic predominance, affecting the interstitium and the tubules, with normal glomeruli. Electron microscopy is usually non-contributory [9].

### 2.5. Treatment

Spontaneous remission can occur, but steroid treatment facilitates remission [9]. Earlier studies showed higher rates of spontaneous remission, with only around 10% of patients requiring steroid therapy. However, more recent studies show that those early findings underestimated the need for chronic steroid therapy due to their short follow-up and management by non-uveitis specialist [9]. The dosing of steroids is usually prednisone or prednisolone 1 to 1.5 mg/kg/day [4], and the duration is dependent on the patient's response.

As in our patient, topical steroids may not be enough when there is significant involvement, especially if there is bilateral, intermediate, or posterior intraocular segment compromise [10]. In one case series, timely corticosteroid therapy significantly improved the levels of beta-2 microglobulin and signs of inflammation in follow-up biopsy in three patients, with a fourth patient that had delayed therapy showing persistent elevations of beta-2 microglobulin and persistent inflammation, with subsequent permanent damage [11].

Despite some evidence of benefit in treatment with corticosteroids, Legendre et al. [12] showed that treatment with corticosteroids was not associated with better kidney outcomes, but it was associated with fewer uveitis relapses. If corticosteroid therapy is not tolerated, patients can be treated with other immunosuppressants, such as cyclosporine, cyclophosphamide, or methotrexate. Mofetil mycophenolate has also been used in the treatment of acute interstitial nephritis [13].

## 3. Conclusion

TINU is a rare disease of apparent immunologic origin, with possible infectious and/or chemical triggers, that affects both the ocular and renal systems. It is more commonly seen in pediatric nephrology, but also seen in some young adults, such as our patient. Our patient had the unusual finding of peripheral retinal vasculitis, which was characterized by alterations such as sheathing, perivascular cuffing, and exudation, along with vascular blockage resulting in capillary non-perfusion, hemorrhages occurring before the retina, and the formation of new blood vessels, not common in TINU patients. Diagnosis usually requires a kidney biopsy in the setting of clinical suspicion, with some laboratory findings including proximal tubular dysfunction and mild proteinuria. Treatment with steroids is more beneficial to avoid relapse of uveitis but does not seem to impact the long-term outcomes when it comes to kidney function. Our patient's kidney function and ocular symptoms improved with short-term corticosteroid therapy, stopped due to medication intolerance.

## Figures and Tables

**Table 1:** Renal function, tubular damage, and eye symptoms of the TINU patient.

	Renal function: creatinine (mg/dL)	Tubular damage: *β*2 microglobulin (mg/L)	Eye symptoms	Snellen VA
04/02/2021	1.93	32.33	Eye redness, blurred vision, photophobia	Right (6/18), left (6/9)
06/07/2021	1.24			
09/05/2022	1.0	2.43	No recurrence	Right (6/7.5), left (6/5)
